# Bed Bug Infestations in an Urban Environment

**DOI:** 10.3201/eid1104.041126

**Published:** 2005-04

**Authors:** Stephen W. Hwang, Tomislav J. Svoboda, Iain J. De Jong, Karl J. Kabasele, Evie Gogosis

**Affiliations:** * St. Michael's Hospital, Toronto, Ontario, Canada;; † University of Toronto, Toronto, Ontario, Canada;; ‡ City of Toronto, Toronto, Ontario, Canada;; § Toronto Public Health, Toronto, Ontario, Canada

**Keywords:** Bed bugs, Parasites, Homeless persons, Urban health, Epidemiology, research

## Abstract

Until recently, bed bugs have been considered uncommon in the industrialized world. This study determined the extent of reemerging bed bug infestations in homeless shelters and other locations in Toronto, Canada. Toronto Public Health documented complaints of bed bug infestations from 46 locations in 2003, most commonly apartments (63%), shelters (15%), and rooming houses (11%). Pest control operators in Toronto (N = 34) reported treating bed bug infestations at 847 locations in 2003, most commonly single-family dwellings (70%), apartments (18%), and shelters (8%). Bed bug infestations were reported at 20 (31%) of 65 homeless shelters. At 1 affected shelter, 4% of residents reported having bed bug bites. Bed bug infestations can have an adverse effect on health and quality of life in the general population, particularly among homeless persons living in shelters.

## Abstract 2

Bed bug infestations adversely affect health and quality of life, particularly among persons living in homeless shelters.

The common bed bug (*Cimex lectularius*) is a wingless, red-brown, blood-sucking insect that grows up to 7 mm in length and has a lifespan from 4 months up to 1 year (Figure 1) (*1*). Bed bugs hide in cracks and crevices in beds, wooden furniture, floors, and walls during the daytime and emerge at night to feed on their preferred host, humans.

**Figure 1:**
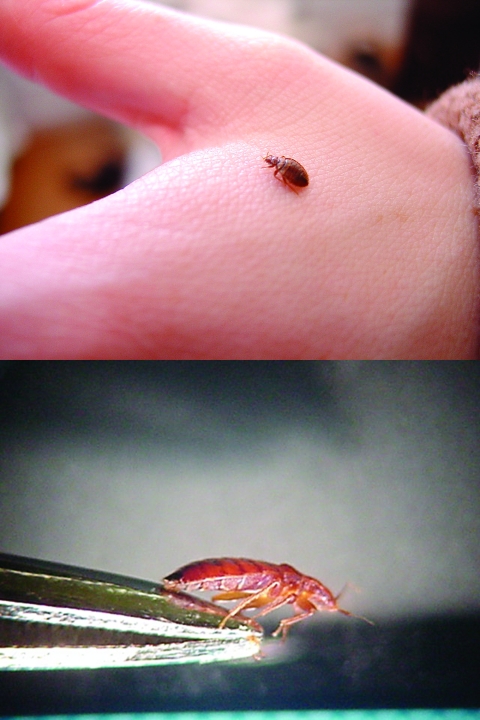
Dorsal and lateral views of a bed bug (*Cimex lectularius*).

The common bed bug is found worldwide. Infestations are common in the developing world, occurring in settings of unsanitary living conditions and severe crowding (*2**,**3*). In North America and Western Europe, bed bug infestations became rare during the second half of the 20th century and have been viewed as a condition that occurs in travelers returning from developing countries (*4*). However, anecdotal reports suggest that bed bugs are increasingly common in the United States, Canada, and the United Kingdom (*5**–**10*). This study was conducted to document the magnitude and adverse effects of bed bug infestations in homeless shelters and other locations in Toronto. Ethical and scientific approval was obtained from Toronto Public Health and the St. Michael's Hospital Research Ethics Board. Shelter staff and residents gave informed consent before participation.

## Methods

### Toronto Public Health and Pest Control Operators Survey

The log of telephone calls made in 2003 to Toronto Public Health was reviewed to identify calls related to bed bug infestations, the types of locations affected, and the regions of the city where infestations were reported. Toronto is divided into 4 public health regions (north, east, south, and west); the south region includes the downtown core of the city. The population of each region was determined from 2001 census data and ranged from 500,000 to 700,000.

A telephone survey of all pest control operators listed in the Toronto telephone directory was conducted using a structured interview. The survey documented the number of bed bug–related calls received, the number of treatments provided by pest control operators in 2003, and the types of insecticides used to treat bed bug infestations. To protect the confidentiality of persons and establishments affected by bed bugs, we asked each pest control operator to report the number of different locations treated for bed bug infestations by general type (e.g., apartment, single-family dwelling, shelter) and not by specific name or address.

### Survey of Homeless Shelter Staff

A telephone interview of the director or supervisor at each homeless shelter in Toronto was conducted to determine which shelters had experienced bed bug infestations. Interviewees were assured that the information they provided would be reported in a way that would not identify their shelter. At affected shelters, follow-up, in-person interviews were conducted with staff from December 2003 to May 2004. A predefined strategy was used to select shelter managers, front-line staff, and healthcare professionals for interviews. The questionnaire included items on time course, manifestations, and extent of the infestation; control measures undertaken; and effects of the infestation on shelter residents and staff. Bed bug infestations were considered confirmed if an entomologist or pest control operator identified a specimen collected at the shelter as *C. lectularius*. Infestations were considered probable if shelter staff reported resident complaints consistent with bed bug infestations.

### Homeless Shelter Resident Survey

As part of a separate study of bacterial colonization among shelter residents, a sample of 243 residents at 1 shelter affected by bed bugs was surveyed in July and August 2003. Participants were selected at random from among persons registered at the shelter, and 80% of those contacted agreed to participate. Participants were asked if they currently had any skin-related illness, injury, or condition, and if so, what type. We obtained permission from the principal investigator of this study to review participant responses to determine the prevalence of self-reported bed bug bites (G. Bargh, pers. comm.).

## Results

### Calls to Toronto Public Health

In 2003, Toronto Public Health received insect-related calls from 82 different street addresses; 46 were complaints of bed bug infestations, 11 were requests for information about bed bugs, and 25 were unrelated to bed bugs. The 46 separate locations where infestations were reported are shown in Table 1. In response to these calls, public health staff spent a total of 27 hours providing information, and health inspectors spent a total of 78 hours conducting site visits to confirm complaints and offer assistance. More complaints of infestations were received in the last 6 months (31 calls) than in the first 6 months (15 calls) of 2003. In the south region, which includes the downtown core of the city, 4.7 complaint calls were received per 100,000 population; this rate was 6.1 times higher (95% confidence intervals [CI] 3.3–11.4) than the rate in the rest of the city. A total of 32 complaints (70%) were from locations in the south region.

**Table 1:** Reports of bed bug infestations in Toronto, 2003

Type of location	Calls to pest control operators			Calls to Toronto Public Health
	No. locations treated (%)*	No. treatments (%)	Mean no. treatments per location	No. locations (%)
Single-family dwelling	588 (70)	641 (49)	1.1	2 (4)
Apartment unit	155 (18)	297 (23)	1.9	29 (63)
Homeless shelter	68 (8)	218 (17)	3.2	8 (17)
Hotel	19 (2)	96 (7)	5.1	1 (2)
Rooming house	6 (0.7)	16 (1)	2.7	5 (11)
Community center	5 (0.5)	5 (0.4)	1.0	1 (2)†
University dormitory	4 (0.5)	36 (3)	9.0	0 (0)
Restaurant	1 (0.1)	1 (0.1)	1.0	0 (0)
Other residential institution	1 (0.1)	5 (0.4)	5.0	0 (0)
Total	847 (100)	1,315 (100)	1.6	46 (100)

*Figures in this column may reflect some double counting of locations (see details in Methods section). †Infestation located at the clothing bank in a community center.

### Survey of Pest Control Operators

We interviewed 34 (89%) of 38 pest control operators listed in the Toronto phonebook; 20 (59%) had provided treatments for bed bugs in 2003. Among these pest control operators, 17 (85%) of 20 reported that they had received an increased number of calls related to bed bugs and had provided more treatments for bed bugs in 2003 than in 2002. The number of locations treated by pest control operators in 2003 and the number of treatments required are shown in Table 1. The mean number of treatments required per affected location was highest at dormitories, hotels, homeless shelters, and rooming houses.

### Homeless Shelter Staff and Resident Survey

**Figure 2:**
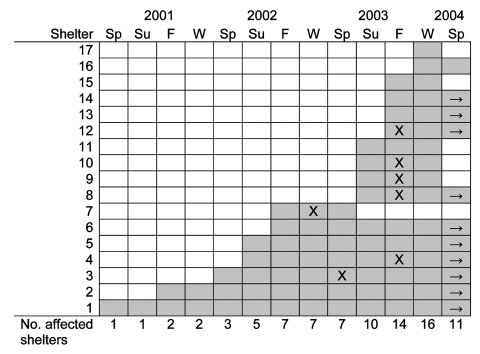
Time course of bed bug infestations in homeless shelters in Toronto. Shaded boxes indicate periods of infestation, X indicates peak period (if reported), and → indicates infestation ongoing as of spring 2004. Sp, spring (March, April, May); Su, summer (June, July, August); F, fall (September, October, November); W, winter (December, January, February).

We contacted all 65 homeless shelters in Toronto and found that 20 (31%) shelters reported previous or current bed bug infestations. Permission was obtained to survey staff at 17 (85%) of 20 affected shelters. Three shelters reporting bed bug infestations either declined or did not respond to our request to interview shelter staff to obtain further information. Forty-three staff members (1–9 per shelter) were interviewed. The time course of the infestation at these shelters is shown in Figure 2. The number of shelters with active bed bug infestations increased steadily from spring 2001 to winter 2003 and then declined. At the end of spring 2004, infestations persisted at 10 shelters. These 10 shelters accounted for 30% of the total shelter bed capacity in the city of Toronto.

At the 17 affected shelters, staff became aware of bed bugs through resident complaints (16 shelters, 94%), visual sightings (14 shelters, 82%), and bite marks on residents (13 shelters, 76%). Staff at 1 shelter (6%) reported that a healthcare provider alerted them to the infestation. A pest control operator or entomologist made a positive identification of the common bed bug at 13 affected shelters (76%). At 3 shelters (18%), residents contacted Toronto Public Health and requested a visit by a health inspector. At 5 locations (29%), shelter staff complained of bed bug bites. Of 243 residents interviewed at an affected shelter in the summer of 2003, 9 persons (4%) had a skin condition that they described as bed bug bites.

The affected locations at homeless shelters and the chemical control measures implemented are shown in Table 2. Professional pest control operators applied insecticides (most commonly, cyfluthrin, bendiocarb, propoxur, and permethrin) at 12 shelters (71%). Shelter staff applied insecticides (most commonly, pyrethrin and propoxur) at 13 shelters (76%).

**Table 2:** Locations at homeless shelters affected by bed bugs and chemical and environmental control measures implemented

Locations and control measures	No. shelters (%), N = 17
Affected locations	
Sleeping rooms	15 (88)
Bed or bed frames	15 (88)
Mattresses	13 (76)
Sheets	13 (76)
Floorboards or walls	9 (53)
Lockers	3 (18)
Other*	11 (65)
Nonsleeping rooms†	11 (65)
Chemical control measures (insecticides)	
Spot treatment only	4 (24)
Treatment of affected rooms	5 (29)
Treatment of entire building‡	8 (47)
All beds dismantled and treated	5 (29)
Environmental control measures	
Residents encouraged to shower and wash belongings	17 (100)
Increased room inspections to detect infestations	13 (76)
Ripped or torn mattresses discarded	8 (47)
Limits on amount of personal belongings	8 (47)
Beds and bedding steam cleaned and vacuumed	6 (35)
Building renovations§	6 (35)
Adhesive boards on the legs of beds to trap bugs	4 (24)
Replacing wooden beds with steel beds	3 (18)

*Other areas consisted of personal belongings, light fixtures, electrical switches and plugs, baseboards, carpeting, and other furniture. †Affected nonsleeping rooms were the lounge, cafeteria, intake office, or storage room. ‡Treatment of the entire building entailed closing the shelter for 6 to 72 hours. §See text for details.

Shelters implemented a number of environmental control measures (Table 2). To control bed bugs, 6 shelters (35%) made substantial building repairs, including removing floorboards, baseboards, or wood trim; replacing carpets; sealing floor cracks; and painting wooden floors. Two shelters (12%) installed additional washers and dryers to deal with increased laundry demands. The total cost incurred for bed bug control efforts at affected shelters was U.S.$150–$15,000, with a mean of U.S.$3,085 per affected shelter.

## Discussion

This study delineates the broad extent of a recent resurgence of bed bug infestations in an urban environment. In light of anecdotal reports from other localities (*5**–**10*), we believe that this phenomenon is likely occurring in cities across North America and Europe. The reasons for this resurgence are unknown, although some reports have suggested a role for increasing world travel, reluctance to use insecticides because of concerns regarding toxicity, and insecticide resistance (*9**,**10*). Although initial reports in Toronto indicated that bed bug infestations were occurring primarily in homeless shelters, our study showed that bed bugs are found in a wide variety of locations in the urban environment, including single-family dwellings, apartments, and rooming houses.

Data from public health officials and pest control operators provided markedly different perspectives on the extent and localization of infestations. This difference may reflect a tendency for persons experiencing bed bug infestations in single-family dwellings to rely on pest control operators, whereas apartment dwellers and homeless shelter staff may be more likely to contact public health authorities. The Toronto experience indicates that these calls place a substantial time demand on public health personnel, who in many cities are already struggling with limited resources.

Our data suggest that bed bugs can spread from shelter to shelter, presumably transported in the personal belongings of residents. At an affected homeless shelter, 4% of residents reported having bed bug bites; given the constant turnover of shelter residents, bed bugs could potentially affect a large number of homeless people over the course of a year. In our clinical experience, homeless persons with bed bug bites suffer a substantial degree of emotional distress.

Infestations in shelters are difficult and costly to eradicate. Our observation of a high mean number of pest control treatments per affected location (Table 1) points to the likelihood that infestations will be difficult to control in other communal living settings and in hotels. The pest control literature emphasizes the importance of combining insecticide treatments with environmental measures such as daily laundering of bed linens, vacuuming rooms, and steam cleaning and vacuuming mattresses. Bed bugs can be destroyed by freezing or by heat treatments at temperatures >50°C, but these methods are inconvenient to implement (*9**,**10*)

Bed bug bites can result in clinical manifestations; the most common are small clusters of extremely pruritic, erythematous papules or wheals that represent repeated feedings by a single bed bug (*1*). Less common but more severe manifestations include grouped vesicles, giant urticaria, and hemorrhagic bullous eruptions (*11*). Bites should be managed symptomatically with topical emollients, topical corticosteroids, oral antihistamines, or some combination of these treatments.

Health professionals should be aware of this reemerging urban pest to facilitate prompt diagnosis of affected patients and treatment of the underlying environmental infestation. Bed bug bites must be differentiated from scabies, body lice, and other insect bites. Diagnostic clues include clustering and timing of bed bug bites. Unlike body lice, bed bugs are rarely found on affected persons or their clothing, and persons with good personal hygiene who enter an infested area are likely to be bitten.

Although bed bugs could theoretically act as a disease vector, as is the case with body lice, which transmit *Bartonella quintana* (the causal agent of trench fever) among homeless persons (*12*), bed bugs have never been shown to transmit disease in vivo (*13*). Hepatitis B viral DNA can be detected in bed bugs up to 6 weeks after they feed on infectious blood, but no transmission of hepatitis B infection was found in a chimpanzee model (*14**–**19*). Transmission of hepatitis C is unlikely, since hepatitis C viral RNA is not detectable in bed bugs after an infectious blood meal (*18*). Live HIV can be recovered from bed bugs up to 1 hour after they feed on infected blood, but no epidemiologic evidence for HIV transmission by this route exists (*20**–**22*).

This study has certain limitations. Shelter data were based on self-reports from staff at affected shelters. Although we obtained data from multiple informants at each shelter when possible, we did not independently verify the accuracy of these reports. Affected shelters represented 30% of shelter beds in Toronto, but our methods did not determine how many rooms or beds within each shelter were affected. Shelter residents' reports of having bed bug bites were not independently confirmed, and some of these persons may have had other types of insect bites or delusions of parasitosis. The method we used to survey pest control operators may have resulted in double-counting locations that obtained treatments for bed bugs from >1 pest control operator in 2003. As a result, the number of affected locations may be overestimated. Furthermore, the reliability of reports from pest control operators is uncertain. Finally, our results, based on calls to public health and pest control operators, reflect self-initiated complaints from affected locations and therefore do not provide population-based data on the prevalence of bed bug infestations.

In conclusion, our study documents the broad extent of bed bug infestations in an urban environment. This problem could have substantial adverse effect on health and quality of life, particularly among persons who use homeless shelters. Physicians should be aware of the typical dermatologic signs and symptoms of bed bug bites, which may become increasingly common in the future. Further research is needed to determine the geographic extent of the current reemergence of bed bugs in the industrialized world and the prevalence and risk factors for bed bug infestations in the general population, including those living in both congregate and noncongregate housing.
